# Hepatitis E Virus Strains in Rabbits and Evidence of a Closely Related Strain in Humans, France

**DOI:** 10.3201/eid1808.120057

**Published:** 2012-08

**Authors:** Jacques Izopet, Martine Dubois, Stéphane Bertagnoli, Sébastien Lhomme, Stéphane Marchandeau, Samuel Boucher, Nassim Kamar, Florence Abravanel, Jean-Luc Guérin

**Affiliations:** Institut National de la Santé et de la Recherche Médicale, Toulouse, France (J. Izopet, M. Dubois, S. Lhomme, N. Kamar, F. Abravanel);; Faculté de Médecine Toulouse-Purpan, Université Toulouse III Paul-Sabatier, Toulouse (J. Izopet, S. Lhomme, N. Kamar, F. Abravanel);; Le Centre Hospitalier Universitaire de Toulouse, Toulouse (J. Izopet, M. Dubois, S. Lhomme, N. Kamar, F. Abravanel);; École Nationale Vétérinaire de Toulouse, Toulouse (S. Bertagnoli, J.-L. Guérin);; Office National de la Chasse et de la Faune Sauvage, Nantes, France (S. Marchandeau);; and Labovet, Les Herbiers, France (S. Boucher )

**Keywords:** Hepatitis E virus, rabbit, zoonoses, viruses, Europe, France, transmission, human

## Abstract

Hepatitis E virus (HEV) strains from rabbits indicate that these mammals may be a reservoir for HEVs that cause infection in humans. To determine HEV prevalence in rabbits and the strains’ genetic characteristics, we tested bile, liver, and additional samples from farmed and wild rabbits in France. We detected HEV RNA in 7% (14/200) of bile samples from farmed rabbits (in 2009) and in 23% (47/205) of liver samples from wild rabbits (in 2007–2010). Full-length genomic sequences indicated that all rabbit strains belonged to the same clade (nucleotide sequences 72.2%–78.2% identical to HEV genotypes 1–4). Comparison with HEV sequences of human strains and reference sequences identified a human strain closely related to rabbit strain HEV. We found a 93-nt insertion in the X domain of open reading frame 1 of the human strain and all rabbit HEV strains. These findings indicate that the host range of HEV in Europe is expanding and that zoonotic transmission of HEV from rabbits is possible.

## Abstract 2

The host range of HEV in Europe is expanding, and zoonotic transmission of HEV from rabbits is possible.

Hepatitis E virus (HEV) is a major cause of acute hepatitis in many developing countries in Asia and Africa, where it is transmitted by the fecal–oral route because of poor sanitation practices (*1*). Acute hepatitis E is also increasingly reported in industrialized countries, where the transmission is mainly zoonotic (*2*). The initial discovery of HEV transmission from domestic pigs (*3*) has been followed by evidence that other mammals, such as wild boars and deer, are also potential reservoirs of HEV (*4*). Although the course of HEV infection is generally self-limiting and asymptomatic (or symptomatic with acute hepatitis), fulminant hepatitis can occur in pregnant women and in persons with underlying liver disease (*5**–**7*). HEV infections can also become chronic in immunocompromised patients, such as recipients of solid-organ transplants (*8**–**10*), those with hematologic diseases (*11**,**12*), and patients infected with HIV (*13**–**15*).

HEV, genus *Hepevirus*, family *Hepeviridae*, is a positive-sense, single-stranded, nonenveloped RNA virus (*16*). The HEV genome is ≈7.2 kb long and contains 3 open reading frames (ORFs) as well as 5′ and 3′ untranslated regions: ORF1 encodes nonstructural proteins, ORF2 encodes the capsid protein, and ORF3 encodes a small phosphoprotein. Phylogenetic analysis of HEV sequences has led to the recognition of 4 major genotypes that infect mammals from a variety of species. HEV1 and HEV2 are restricted to humans and transmitted through contaminated water in developing countries. HEV3 and HEV4 infect humans, pigs, and other mammals and are responsible for sporadic cases of hepatitis E in developing and industrialized countries (*2*). HEV3 is distributed worldwide, whereas HEV4 largely is found in Asia. Although HEV3 and HEV4 infections have been linked to the consumption of raw or undercooked meats, such as pig liver sausages or game meats (*17**,**18*), the full spectrum of animals that are reservoirs of HEV is still unknown.

Recent studies have characterized new HEV genotypes in isolates from rats in Germany (*19*), wild boars in Japan (*20*), and farmed rabbits in the People’s Republic of China (*21**,**22*). Because the potential risk for zoonotic transmission of HEV from rabbits in France is unknown, and cases of autochthonous hepatitis E are commonly reported in this country (*23**,**24*), we investigated the prevalence of HEV in farmed and wild rabbits. We also looked for a genetic link between HEV strains circulating in rabbits and HEV strains circulating in humans in France.

## Materials and Methods

### Specimens from Farmed Rabbits

Bile specimens (n = 200) were collected in September 2009 from rabbits raised on 20 farms in western France, in the departments of Maine et Loire (n = 6), Vendée (n = 6), Deux-Sèvres (n = 4), Calvados (n = 2), and Loire Atlantique (n = 2), the main geographic areas of rabbit farming in France. We sampled 10 rabbits from each farm when they were slaughtered at 70–90 days of age (Table 1). All rabbits were healthy and intended for human consumption. All samples were immediately stored at −80°C.

**Table 1:** Detection of hepatitis E virus RNA in farmed and wild rabbits, France

Source	Location/department	No. tested	No. (%) HEV RNA positive
Farmed rabbits			
F20	Calvados	10	0
F4	Calvados	10	1 (10)
F6	Deux-Sèvres	10	1 (10)
F7	Deux-Sèvres	10	1 (10)
F12	Deux-Sèvres	10	0
F16	Deux-Sèvres	10	0
F3	Loire Atlantique	10	2 (20)
F8	Loire Atlantique	10	0
F1	Maine et Loire	10	5 (50)
F5	Maine et Loire	10	1 (10)
F10	Maine et Loire	10	0
F15	Maine et Loire	10	0
F17	Maine et Loire	10	0
F19	Maine et Loire	10	0
F2	Vendée	10	3 (30)
F9	Vendée	10	0
F11	Vendée	10	0
F13	Vendée	10	0
F14	Vendée	10	0
F18	Vendée	10	0
Wild rabbits			
W9	Charentes	26	3 (12)
W5	Deux-Sèvres	44	13 (30)
W14	Deux-Sèvres	3	0
W2	Dordogne	5	3 (60)
W6	Dordogne	8	3 (38)
W8	Dordogne	4	1 (25)
W11	Dordogne	1	0
W12	Dordogne	5	0
W15	Dordogne	4	0
W16	Dordogne	17	0
W1	Finistère	10	10 (100)
W4	Finistère	10	4 (40)
W7	Finistère	15	4 (27)
W3	Haute-Garonne	12	6 (50)
W13	Loire Atlantique	11	0
W18	Loire Atlantique	1	0
W10	Morbihan	10	0
W17	Pyrénées Orientales	19	0

*F, farm; W, warren.

### Specimens from Wild Rabbits

Liver specimens (n = 205) were collected during September 2007–November 2010 from 18 populations of wild rabbits, established in warrens; each population was considered epidemiologically independent. The populations were located in several departments of mainland France: Dordogne (n = 7), Finistère (n = 3), Deux-Sèvres (n = 2), Loire-Atlantique (n = 2), Haute-Garonne (n = 1), Charentes (n = 1), Morbihan (n = 1), and Pyrénées-Orientales (n = 1) (Table 1). The number of rabbits sampled in a given warren ranged from 1 to 44. They were >6 months of age, apparently healthy, and intended for human consumption. Each rabbit was eviscerated within a few hours of its death, and a sample of its liver was taken and immediately frozen at −80°C. Necropsies were performed on a group of 12 rabbits from the same warren in Haute-Garonne (W3), and samples of their intestine and cecum were taken, in addition to samples from the liver.

### Specimens from Humans

Serum specimens were collected from immunocompetent and immunocompromised patients who had received a diagnosis of hepatitis E from the department of virology at Toulouse University Hospital. All samples were stored at −80°C (*23**,**24*).

### RNA Extraction

Samples (140 μL of rabbit bile and 50 mg of liver, intestine, and cecum) were disrupted with TRIzol (Invitrogen, Saint Aubin, France). RNA was extracted with QIAamp Viral RNA Mini Kits (QIAGEN, Courtaboeuf, France).

### Real-time Reverse Transcription PCR

We used 1-step real-time reverse transcription PCR on the Light Cycler 480 instrument (Roche Diagnostics, Meylan, France) to amplify a 70-bp fragment. The primers and probes targeted the ORF3 region: forward primer HEVORF3-S: 5′-GGTGGTTTCTGGGGTGAC-3′, reverse primer HEVORF3-AS: 5′AGGGGTTGGTTGGATGAA-3′, and probe 5′-Fam-TGATTCTCAGCCCTTCGC-Tamra-3′ (*25*). Each 50-μL reaction mix contained 1 μL of SuperScript III Platinum One-Step Quantitative RT-PCR System (Invitrogen), 15 μL of RNA, primers (200 nmol/L) and probes (150 nmol/L), and 40 U of RNase Out (Invitrogen). Reverse transcription was carried out at 50°C for 15 min, followed by denaturation at 95°C for 1 min. DNA was amplified with 50 PCR cycles at 95°C (20 s) and 58°C (40 s). HEV RNA was quantified by using a transcribed RNA standard constructed from a genotype 3f HEV strain (GenBank accession no. EU495148). The limit of detection was 100 copies/mL.

### DNA Sequencing

Two fragments, one within ORF2 (189 bp) and the other within ORF1, encompassing the hypervariable region and X domain (≈1,400 bp), were amplified and sequenced in both directions by the dideoxy chain termination method (PRISM Ready Reaction Ampli Taq Fs and Dye Deoxy primers; Applied Biosystems, Paris, France) on an ABI 3130XL capillary DNA analyzer (Applied Biosystems, Foster City, CA, USA). The primers used for the ORF2 fragment were the following: forward primer HEVORF2-S: 5′-GACAGAATTRATTTCGTCGGCTGG-3′ and reverse primer HEVORF2-AS: 5′-TGYTGGTTRTCATAATCCTG-3′. The primers used for the ORF1 fragment were the following: forward primer HEVORF1-S: 5′-TGACGGCYACYGTKGARCTTG-3′ and reverse primer HEVORF1-AS: 5′-ACATCRACATCCCCCTGYTGTATRGA-3′.

The whole genomes of 2 rabbit strains (W1–11 and W7–57) and 1 human strain (TLS-18516-human) were amplified by overlapping RT-PCR. The primers are listed in Table 2.

**Table 2:** Primers used to amplify and sequence the whole genome of HEV strains, France*

Fragment size, bp	Nucleotide position†	Primer	Sequence, 5′ → 3′
225	15–238	Sense	ATGTGGTCGATGCCATGGAGGCCCA
		Antisense	CTCATTATGTATAACACGTTGAATAG
2,900	152–3937	Sense	AGACAGATATTCTTATCAATTTAATGCAACCCCGC
		Antisense	GCCGCAAGTAACACGGGCGGCCGTGTGAGGTGTGAA
2,000	3207–5212	Sense	AAGTCTAGGTCTATACAGCAGGG
		Antisense	GCCGGTGGCGCGGGCAGCATAGGCA
2,160	5060–7208	Sense	AATGTYGCYCAGGTYTGTG
		Antisense	TTTTTTTTTTTTCCYGGGRGCGC

*HEV, hepatitis E virus. †Nucleotide position refers to Burma strain, M73218.

### Phylogenetic Analysis

The genotype was determined by using reference strains as previously described (*26*). Phylogenetic analyses were performed with genotype information on reference sequences based on the HEV classification proposed by Lu et al. (*27*). Sequences were aligned by using ClustalW (MEGA5, www.megasoftware.net; BioEdit version 7.0, www.mbio.ncsu.edu/bioedit/bioedit). Phylogenetic trees were created by the neighbor-joining (Kimura 2-parameter) method with a bootstrap of 1,000 replicates.

The 2 partial sequences of ORF1 and the 5 full-length sequences reported in this study have been deposited in GenBank. The accession numbers are JQ013789 and JQ013790 for ORF1, and JQ013791 to JQ013795 for the full-length sequences of W1–11, W7–57, TLS- 18516-human, TR19 (genotype 3c), and TR02 (genotype 3e), respectively.

## Results

### HEV RNA

All bile specimens from the 200 farmed rabbits and the liver specimens from the 205 wild rabbits were tested for HEV RNA (Table 1). Samples from 7 farms (35%) and 9 warrens (50%) tested positive for HEV RNA. HEV RNA was found in a 14 bile samples (7%) from farmed rabbits. The median HEV RNA concentration in the bile samples was 2.3 × 10^7^ copies/mL (range 100 copies/mL–10^9^ copies/mL). A total of 47 liver samples (23%) from wild rabbits were positive for HEV RNA; median HEV RNA concentration was 1.9 × 10^6^ copies/g (range 1,400 copies/g–5.8 × 10^7^ copies/g).

We tested the liver, intestine, and cecum samples from 12 wild rabbits from the same warren (W3) in triplicate to obtain a clear picture of the tissue distribution of HEV in infected rabbits. HEV RNA was detected in all the tissues from 4 rabbits (nos. 4, 7, 9, 12), in the liver and intestine of 1 rabbit (no. 5), and in the liver only of 1 rabbit (no. 6) (Table 3). The virus loads in the liver (mean 4.8 log copies/g), intestine (mean 4.0 log copies/g), and cecum (mean 3.6 log copies/g) were not significantly different.

**Table 3:** Detection of HEV RNA by real time RT-PCR in the 12 wild rabbits from warren W3 according to tissue sampled, France*

Rabbit no.	Liver			Intestine			Cecum	
	No. positive	Mean viral load†		No. positive	Mean viral load†		No. positive	Mean viral load†
1	0	<LOD		0	<LOD		0	<LOD
2	0	<LOD		0	<LOD		0	<LOD
3	0	<LOD		0	<LOD		0	<LOD
4	3	7.3		3	5.3		2	3.4
5	3	3.7		1	3.1		0	<LOD
6	3	4.4		0	<LOD		0	<LOD
7	1	3.1		2	3.6		1	3.8
8	0	<LOD		0	<LOD		0	<LOD
9	3	6.6		3	5.2		3	4.6
10	0	<LOD		0	<LOD		0	<LOD
11	0	<LOD		0	<LOD		0	<LOD
12	3	3.7		2	3.6		1	3.8

*Three samples were obtained of each tissue type from each rabbit. HEV, hepatitis E virus; RT-PCR, reverse transcription PCR; LOD, limit of detection. †log copies/g.

### ORF2 Sequences

**Figure 1:**
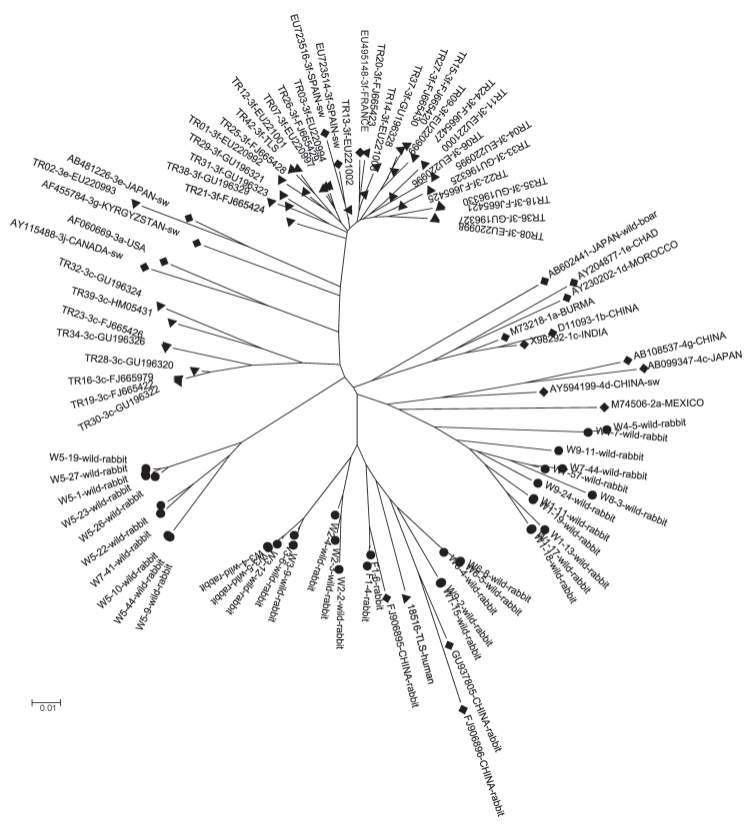
Phylogenetic tree for the 189-bp sequence of open reading frame 2 of the capsid gene of rabbit hepatitis E virus (HEV) strains (circles), human strains circulating in France (triangles), and reference strains (diamonds). GenBank accession numbers are shown for each HEV strain used in the phylogenetic analysis. Scale bar indicates nucleotide substitutions per site.

Phylogenetic analyses, conducted on the basis of a 189-nt fragment within ORF2 of the 37 HEV strains from rabbits, HEV3 strains from humans circulating in France, and HEV reference sequences (HEV1, HEV2, HEV3, HEV4, rabbit HEV, rat HEV, wild-boar HEV) indicated that the 37 new ORF2 sequences from rabbit HEVs were clustered. One cluster contained 3 ORF2 sequences from previously characterized HEV from farmed rabbits from China, 2 ORF2 sequences from HEVs from farmed rabbits in France, and 13 ORF2 sequences from HEVs from wild rabbits in France (Figure 1). This cluster also contained an ORF2 sequence from a strain from a person in France (TLS-18516-human) (Figure 1). This strain was found in a serum sample from a 46-year-old man with an elevated alanine aminotransferase level (400 IU/L, reference <35 IU/L).

### ORF1 Sequences

Phylogenetic analysis based on a 1,400-nt fragment within ORF1, indicated that the ORF1 sequences from HEV strains from rabbits in France (n = 4) or China (n = 3) and the ORF1 sequence from the human strain TLS-18516-human formed a distinct genetic group among sequences of HEV genotypes 1–4 (data not shown). The cluster of rabbit HEV sequences was also distinct from the HEV sequences from wild-boar and rat HEV genotypes that were characterized recently.

Comparison of the ORF1 sequences from rabbit HEV strains with reference ORF1 sequences from HEV genotypes 1–4 showed an insertion of 93 nt in the X domain of the ORF1 of all the rabbit HEV strains. This insertion was also found in the TLS-18516-human strain. The deduced amino acid sequences corresponding to this insertion, located between amino acids 938 and 939 (Burmese strain, M73218), were not very similar, except for 2 conserved amino acids at the C-terminal end.

### Genome Sequences

**Figure 2:**
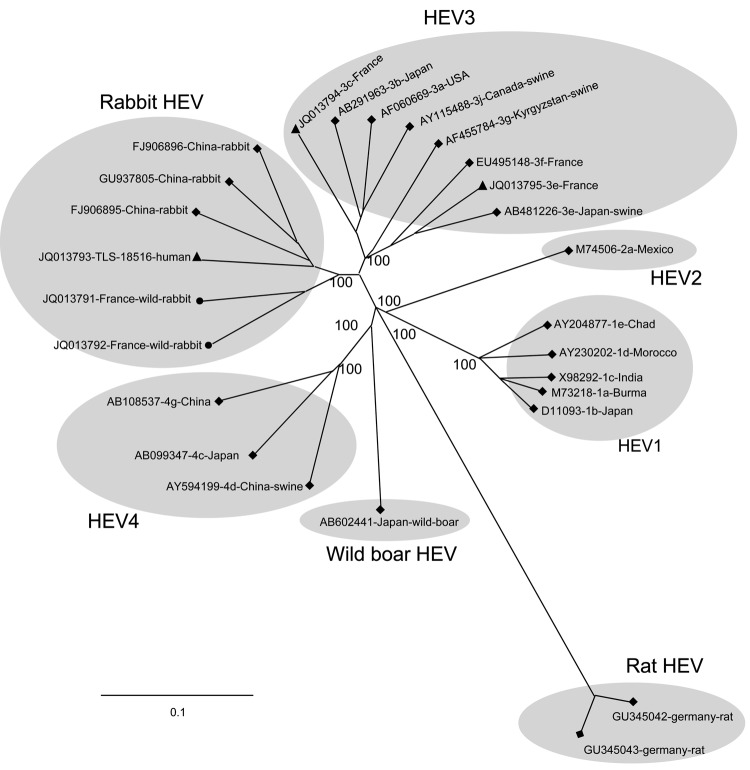
Phylogenetic tree based on full-length sequences of hepatitis E virus (HEV) rabbit strains (circles), the human strain TLS-18516 (triangles) and reference strains (diamonds). GenBank accession numbers are shown for each HEV strain used in the phylogenetic analysis. Scale bar indicates nucleotide substitutions per site.

We obtained the full-length genomic sequences of HEV strains from 2 wild rabbits in France and the TLS-18516-human strain. The phylogenetic tree, constructed by the neighbor-joining method using the full-length genomic sequences (including the sequences of genotypes 3f, 3e, and 3c, which were circulating in France), revealed that the HEV genomes from the rabbit strains and the TLS-18516-human strain belonged to the same clade. This clade was clearly separated from genotypes 1–4, found in other mammals and from the new HEV genotypes found in wild boars and rats (Figure 2).

The length of the rabbit strain W1–11 genome, excluding the poly(A) tract at the 3′ terminus, was 7,262 nt. The length of the rabbit strain W7–57 was 7,231 nt, and that of the TLS-18516-human strain was 7,259 nt. The nucleotide sequences of the rabbit strains and the TLS-18516-human strain were 80.3%–85% identical (Table 4). The nucleotide sequences of the rabbit or TLS-18516-human strains were 76.1% to 78.2% identical to those of genotype 3, 72.7% to 73.7% identical to those of genotype 1, 72.2% to 73.5% identical to those of genotype 2, and 72.9% to 74.9% identical to those of genotype 4. These comparisons therefore indicate that the sequences of the rabbit HEV strains and the TLS-18516-human strain are distinct from all known strains of HEV genotypes 1–4 and from the newly described HEV genotypes from wild boars and rats.

**Table 4:** Percent identities of full-length sequences among HEV strains from rabbits and other HEV strains, France*

Virus strain	HEV strains, % identity				
	TLS-18516	HEV1	HEV2	HEV3	HEV4
GU937805-China-rabbit	85.0	73.0–73.7	72.2	76.3–78.2	72.9–73.9
W7–57-wild-rabbit	80.3	72.7–73.4	73.5	76.2–77.6	73.2–73.6
W1–11-wild-rabbit	80.6	73.2–73.7	73.4	76.1–78.0	74.2–74.9

*HEV, hepatitis E virus; HEV1 (M73218–1a-Burma, D11093–1b-Japan, X98292–1c-India, AY204877–1e-Chad, AY230202–1d-Morocco); HEV2 (M774506–2a-Mexico) ; HEV3 (AF060669–3a-USA, AY115488–3j-Canada-Sw, AB291963–3b-Japan, TLS-TR19–3c, TLS-TR02–3e, AB481226–3e-Japan-Sw,EU495148–3f-France,AF455784–3g-kyrgyzstan-Sw); HEV4 (AB099347–4c-Japan, AB108537–4g-China, AY594199–4d-China-Sw).

## Discussion

We found that farmed and wild rabbits in France are naturally infected with HEV. We also characterized a human HEV strain that is closely related to rabbit HEV strains; this finding thus supports the potential of zoonotic transmission from rabbits to humans.

The HEVs found in farmed rabbits in several geographic areas of China have been identified (*21**,**22*). HEV was also recently found in farmed rabbits in Virginia, USA (*28*). Our study results show that rabbits in Europe are infected with HEV and that some farmed rabbits and wild rabbits in France are infected. We found HEV RNA in 7% of the farmed rabbits and in 23% of the wild rabbits. However, the ages of the rabbits and the tissues tested (bile samples from farmed rabbits and liver samples from wild rabbits) may explain the observed difference in HEV prevalence. Nevertheless, previous studies have shown that the virus loads in liver and bile samples from swine infected with HEV are similar (*29**,**30*). Although the greater prevalence of HEV in wild rabbits could be linked to their older age, we could not test for a relationship between the prevalence of HEV and rabbit age because we did not know the rabbits’ precise ages.

Our analysis of the distribution of HEV in the tissues of infected wild rabbits showed HEV RNA not only in the liver, but also in the intestine and cecum; our analysis also showed that the virus loads from these organs were not significantly different. This finding suggests that extrahepatic sites of HEV replication exist in rabbits, as has been demonstrated for HEV3 in pigs (*31*). However, because the intestine and cecum samples may have been contaminated with blood, our results need to be confirmed in future studies using methods that ensure that tissues other than the liver are not contaminated with blood.

To determine whether rabbits could be a reservoir for viruses that cause human infection, we analyzed partial and complete nucleotide sequences of the rabbit HEV strains and compared these sequences with those of human HEV strains circulating in France. Analysis of ORF2 showed that the sequences from rabbit HEV strains formed clusters, one of which included the sequences of HEV genotypes 2 and 4. The bootstrap values were very low because the fragments analyzed were small. In contrast, phylogenetic analyses based on ORF1 and the full-length genome indicated that all the rabbit strains from China and France belong to the same clade. One human strain, TLS-18516-human, clustered with the rabbit strains and appeared to be somewhat different from the 4 major HEV genotypes found in mammals and the newly described HEV genotypes from rats and wild boars. Although the full-length sequences of the genomes of the rabbit strains and the TLS-18516-human strain are more similar to that of HEV3 than to those of HEV1, HEV2, and HEV4, they do not seem to belong to the established HEV genotype 3 found in humans and swine, as recently suggested (*20**,**32*). Differences in the classification of rabbit HEV could be because the full-length genomic sequences were used as the reference for phylogenetic analyses. Genotype 3 is highly diverse, with 10 identified subtypes (*27*). We included in our analysis the full-length genomes of subtypes 3f, 3c, and 3e, which account for ≈74%, 13%, and 5% of the human and swine HEV strains circulating in France (*26**,**33*). We also included the other full-length genomes representative of HEV3 subtypes, but subtypes 3d, 3h, and 3i are not yet available in GenBank. Our data indicate that the genomes of rabbit HEV strains or TLS-18516-human were <80% identical with HEV3, regardless of which method was used to align the sequences. This finding is compatible with the definition of a new genotype, as previously proposed (*21**,**22*).

We found a 93-nt insertion in the X domain of the ORF1 of the human strain TLS-18516-human and of all the rabbit HEV strains. This insertion, also found in the rabbit HEV strains from China (*34*), is not present in any known strain of HEV genotypes 1–4 or in the new HEV genotypes from rats and wild boars. The X domain corresponds to a macro domain found in the nonstructural polyproteins of several positive-stranded viruses such as togaviruses and coronaviruses (*35**–**37*). This domain can bind polyadenosine diphosphate–ribose regions and could play a role in the replication or transcription of virus RNA. Whether the insertion in the X domain influences the function of the HEV macro domain warrants further investigation. Several determinants, including this insertion, could be essential for specifying the host range, zoonotic transmission, and pathogenesis of rabbit HEV strains (*34*).

What rabbit HEV strains contribute to the epidemiology of hepatitis E in humans is not clear. HEV is endemic to southwestern France, and the annual incidence of locally acquired HEV infections has been estimated as 3.2% (*38**,**39*). A case–control study found that the only factor independently associated with HEV infection was the consumption of game meat, mostly wild boar, deer, and wild rabbit (*23*). However, molecular data from various studies in France indicate that most HEV strains identified belong to genotypes 3f, 3c, or 3e, which are prevalent in pigs and wild boars (*23**,**26**,**40*). A recent study showed the same proportions of genotypes 3f, 3c, and 3e in human and pig populations (*33*). Although this finding could indicate that rabbit HEV strains are less readily transmitted to humans than HEV genotype 3 strains, the primers used for PCR amplification were not specifically designed for rabbit HEV strains. Therefore, the true prevalence of HEV RNA among rabbits and humans may have been underestimated. In addition, genotyping rabbit HEV may have been difficult because reference sequences have become available only recently.

The immunocompetent or immunocompromised status of the patient that became infected with a rabbit HEV strain, as well as the source of his contamination, is unknown because of the lack of medical follow-up. Molecular and epidemiologic studies are needed to determine the prevalence of rabbit HEV strains among immunocompetent and immunocompromised patients.

In conclusion, we have shown that in France, farmed and wild rabbits can be infected with HEV. Phylogenetic analysis, based on full-length genomes and a molecular signature in the X domain of ORF1, indicates that rabbit HEV strains could be a new genotype. Our identification of a human HEV strain that is closely related to rabbit HEV strains reinforces the potential zoonotic risk for infection with this virus. Further studies are needed to demonstrate cross-species transmission directly and to evaluate the contribution of the rabbit reservoir to human HEV infection and disease.
